# National Survey on Support for Nurse Practitioner and Physician Assistant/Associate Postgraduate Fellowship/Residency Programs and Director Compensation

**DOI:** 10.7759/cureus.57919

**Published:** 2024-04-09

**Authors:** Vasco Deon Kidd, Jessica L Horstmann, Shayanna Felder, Kerry Bamrick

**Affiliations:** 1 Orthopedic Surgery, University of California Irvine School of Medicine, Orange, USA; 2 Community Health, Native American Community Clinic, Minneapolis, USA; 3 Accreditation, Consortium for Advanced Practice Providers, Newport Beach, USA

**Keywords:** program support, advanced practice registered nurse, program director salaries, postgraduate training, advanced practice provider, fellowship, residency, physician associate, physician assistant, nurse practitioner

## Abstract

Introduction

Although there has been a significant and steady increase nationwide in the number of physician assistant/associate (PA) and nurse practitioner (NP) postgraduate residency/fellowship programs, there remains a paucity of research related to the level of operational support available in many of these programs to facilitate specialty training. Therefore, the primary aim of this study is to conduct a national survey to gather foundational data regarding advanced practice provider (APP) postgraduate fellowship/residency operational support and program director total compensation data in the United States.

Methodology

A descriptive cross-sectional survey consisting of 27 questions was distributed via email to 336 postgraduate NP, PA, joint NP/PA, and certified nurse-midwifery residency/fellowship programs between November 2023 and December 2023. Frequency tables and descriptive statistics were used to summarize the survey data. Additionally, Spearman's rank correlation coefficients were performed to determine the relationship between the dependent variables and the independent variable.

Results

There was a statistically significant positive relationship between the organization values the postgraduate program and having sufficient administrative time (*r_s_* = 0.342, *p* < 0.001), having adequate support staff (*r_s_* = 0.340, *p* < 0.001), and being fairly compensated (*r_s_* = 0.356, *p* < 0.001) for program roles and responsibilities. Moreover, slightly less than half of respondents reported having adequate support staff and sufficient administrative time to address program responsibilities. Only 50% of respondents believed they were fairly compensated for their position. Respondents moderately agreed that their organization values the postgraduate training program (M = 4.00, SD = 1.00).

Conclusions

The results of this study highlight the perceptions of postgraduate program directors regarding adequate administrative support and resources to facilitate specialty-specific training for NPs and PAs. Further research is warranted to evaluate the level of operational support needed to develop and sustain postgraduate APP residency/fellowship training programs now and in the future.

## Introduction

Formalized postgraduate training programs for advanced practice providers (APPs), also known as nurse practitioners (NPs) and physician assistants/associates (PAs), are an increasingly prevalent option to facilitate transition-to-practice and enhance specialty-specific knowledge [1-3]. Although not a requirement for entry-level practice, postgraduate residencies/fellowships for licensed PAs have existed since the 1970s and for NPs since 2007. However, only a small percentage of APPs elect to enroll in a postgraduate training program, as evidence supports that entry-level APPs provide safe, high-quality, and cost-effective care without formalized specialty training [4-6]. The range of postgraduate training opportunities available to APPs has grown substantially, as has the demand for more specialized skills to meet the evolving and complex needs of patients. Postgraduate training programs average 12 to 24 months in length with a specific specialty or subspecialty focus, integrating both didactic and clinical components. Programs have the option of multiple accrediting pathways, but the majority remain unaccredited [7].

A considerable body of research exists on describing the programmatic functions and educational characteristics of APP residency/fellowship training programs, including the perspectives of trainees about their postgraduate training experiences [1,2,3,5,8-10]. Yet little is known about program director compensation and whether postgraduate programs have adequate administrative time and support to fulfill various responsibilities. Although the Accreditation Council for Graduate Medical Education has defined minimum specialty-specific program requirements for physician residency and fellowship training in their Guide to the Common Program Requirements (updated 2/15/24 and 3/13/24), the accreditation standards for APP postgraduate programs are less clear about faculty-dedicated time and defined minimum FTE requirements for program personnel. Additionally, there is very little published data on operational support and program director total compensation related to APP postgraduate fellowship/residency programs [1,11]. Providing information on trends will facilitate accreditors, administrators, health policymakers, and researchers in providing guidance to postgraduate programs in the development and sustainability of these training programs. Therefore, the objective of this study is to conduct a national survey to gather foundational data regarding APP postgraduate fellowship/residency operational support and program director compensation data in the United States.

## Materials and methods

This study utilized a cross-sectional research design to obtain information from advanced practice postgraduate training programs in the United States. A web-based survey consisting of 27 items was developed by the study team. Before deployment, the anonymous survey was pretested on a sample of postgraduate program attendees at the annual Consortium for Advanced Practice Providers conference in July 2023. Solicited feedback from the pilot group led to some alterations to survey questions to enhance clarity and improve the order in which questions were asked. The survey consisted of closed and open-ended questions, including free text response options. The survey questionnaire consisted of the following six 5-point Likert scale questions: Q1: Do you have sufficient administrative time to address programmatic responsibilities? Q2: Do you feel you have adequate support staff to complete the APP Postgraduate Training Program responsibilities? Q3: Do you feel you are fairly compensated for your APP Postgraduate Training Program role and responsibilities? Q4: Do you feel your organization values the APP Postgraduate Training Program? Q5: Do you feel your organization values APPs? Q6: Are you satisfied with your work-life balance?

The responses were 1 = strongly disagree, 2 = disagree, 3 = neither agree nor disagree, 4 = agree, and 5 = strongly agree. The purpose of the Likert questions was to determine the relationship between the dependent variables, such as time (Q1), support (Q2), and compensation (Q3) for the postgraduate program role and responsibilities, and the independent variable, organization values for the postgraduate program (Q4).

Using the Consortium for Advanced Practice Providers (www.APPpostgradtraining.com) updated listserv, an email invitation with a link to a voluntary, anonymous, and online survey (Qualtrics Inc., Provo, UT, USA) was distributed to 336 active postgraduate programs. For the purposes of this study, each individual specialty training track was considered a program, even if the training track was embedded within a larger program. The email introduction to the survey contained all the necessary elements of written consent, and submission of the survey indicated the respondents’ consent to participate. Also, within the introductory email was a customized message advising participants to complete the survey once to prevent duplicate responses. Moreover, the survey used a skip logic pattern, allowing participants to skip any question based on responses to preceding questions or if certain questions were not applicable to them.

The study period was from November 20, 2023, through December 31, 2023. Nonrespondents received up to six email reminders over the study period to ensure the highest possible response rate. The average length of time to complete the survey was around eight minutes. No identifying information was obtained from respondents. Survey responses were aggregated, and descriptive statistical analyses were conducted through the online Qualtrics (Qualtrics, Provo, UT) statistical analysis software. When calculating the sample size, we estimated that 180 (54%) or more survey responses were needed to have a confidence level of 95% within a 5% margin of error. The study was approved by the Community Health Center, Inc. Institutional Review Board (approval number: 1207).

Analysis methods

Data were imported into and analyzed using SPSS Statistics version 23 (IBM Corp. Released 2015. IBM SPSS Statistics for Windows, Version 23.0. Armonk, NY: IBM Corp.). Only participants who answered all Q1-Q4 were included in the data analysis. Frequency tables and descriptive statistics were used to summarize the survey data. The normality of the data (Q1-Q4) was examined using the Shapiro-Wilk test. As the data were not normally distributed, the non-parametric method, Spearman's rank correlation coefficients (Field, 2013), was performed to determine the relationship between the dependent variables, such as time (Q1), support (Q2), and compensation (Q3) for the APP postgraduate training program role and responsibilities, and the independent variable, organization value of the APP postgraduate training program (Q4). A p-value less than 0.05 indicated statistical significance [12].

## Results

**Figure 1:**
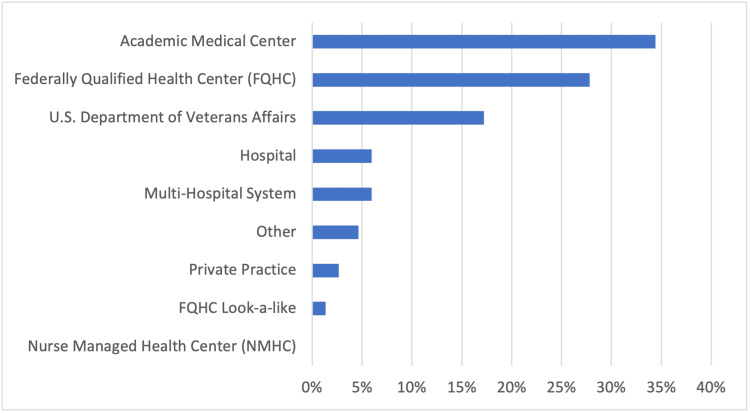
Respondents’ affiliation Total respondents: 151 U.S.: United States

Three hundred and sixty-six postgraduate programs were invited to participate, and 181 postgraduate programs responded to the survey. Twenty-nine participants submitted incomplete surveys, which were excluded from data analysis due to a considerable amount of missing data. The overall response rate was 45.2%. Some respondents opted to skip a question, and the per-question response rates varied. Of those who participated in the survey, 64% (98/152) were NPs, 28% (42/152) were PAs, 5% (7/152) were nonclinical, and 3% (5/152) were physicians. No certified nurse midwives responded to the survey. Respondents were located across multiple institutions, including academic medical centers, 34% (52/151), federally qualified health centers (FQHCs), 28% (42/151), the Department of Veterans Affairs, 17% (26/151), multi-health systems, 6% (9/151), hospitals, 6% (9/151), other (undisclosed location), 5% (7/151), private practice, 3% (4/151), and FQHC lookalikes, 1% (2/151) (Figure 1).

Although FQHC lookalikes function as community-based healthcare centers, they do not receive health center program funding (HRSA.gov). Among respondents, the majority, 48% (72/151), provide postgraduate training to NPs only, while 40% (60/151) train both PAs and NPs, and 13% (19/151) train PAs only. Lastly, although postgraduate programs were dispersed over a broad geographical area, a larger percentage of respondents, 26% (39/152), were from three states (California, New York, and Washington).

Respondents by specialty track

Respondents were asked to indicate the specialty track associated with their postgraduate program trainees, including salary ranges, based on a multi-select question with many options. Given that some respondents were from multi-track programs and may have indicated multiple specialty tracks and salary ranges under a single program, we thought it best to report out an overview of specialty tracks identified in the data. Respondents were associated with the following specialty tracks: acute care, administration specialty, adult gerontology/internal medicine, cardiology, cardiothoracic surgery, cardiothoracic transplant, critical care (adult/pedes), emergency medicine, general surgery, geriatric medicine, hematology/oncology, hospitalist medicine, neonatal critical care, neonatal intensive care, nephrology, neurology, neurosurgery, obstetrics/gynecology, oncology, organ transplant, orthopedic surgery, palliative medicine, pediatric comprehensive cardiac care, pediatric emergency medicine care, pediatric urgent care, general pediatrics, pediatrics acute care, primary care, psychiatry, rural medicine, observation medicine, LGBTQ +health, substance abuse (addiction medicine), trauma surgery, transplant surgery, urgent care, and urology.

Program accreditation

**Figure 2:**
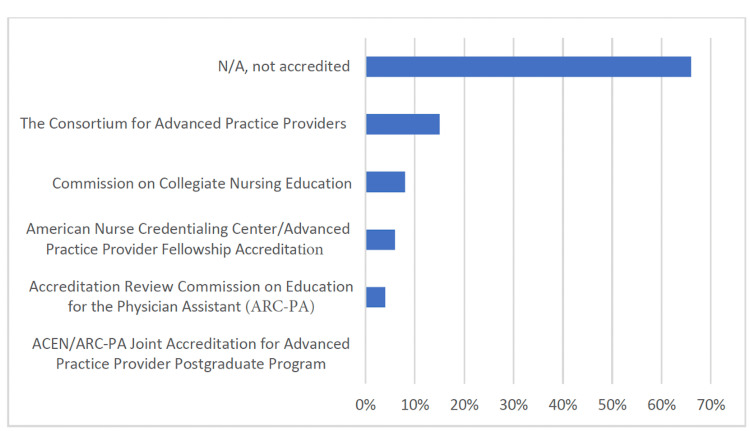
Number of accredited postgraduate programs Total respondents: 149 N/A: not applicable, ACEN/ARC-PA: Accreditation Commission for Education in Nursing/Accreditation Review Commission on Education for the Physician Assistant

Among respondents, the majority, 66% (99/149), of postgraduate programs are not accredited, while 35% (53/149) have achieved accreditation. Among accredited programs, 15% (23/149) of respondents are accredited by the Consortium for Advanced Practice Providers, 8% (12/149) by the Commission on Collegiate Nursing Education, 6% (9/149) by the American Nurse Credentialing Center/Advanced Practice Provider Fellowship Accreditation, and 4% (6/149) by the Accreditation Review Commission on Education for the Physician Assistant (Figure 2).

Program personnel

**Figure 3:**
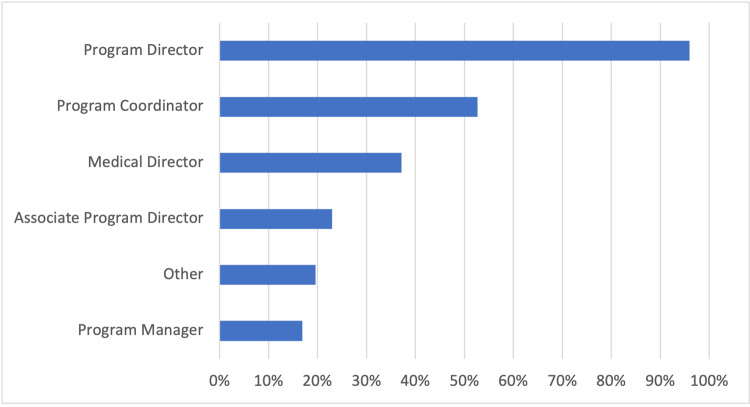
Program personnel Total respondents: 148

The majority of respondents, 96% (142/148), indicated a designated program director; 53% (78/148) a program coordinator; 37% (55/148), a medical director; 23% (34/148) an associate program director; 20% (29/148) other personnel; and 17% (25/148) reported a program manager (Figure 3).

It should be noted that most respondents, 73% (107/146), reported that the program director had five years of clinical experience prior to assuming the role of director. However, only 22% (33/147) of respondents indicated that the current program director had served in their role for more than five years. In terms of full-time equivalent (FTE) administrative support, 52% (68/133) of programs have a total administrative staff FTE of 0.5 or less, whereas 40% (53/133) of programs have 1.0 or more administrative FTE staff. Table 1 is a collection of selected respondent quotes related to administrative support.

**Table 1:** Selected respondent quotes APP: advanced practice provider, FTE: full-time equivalent, NP: nurse practitioner, HRSA: Health Resources and Services Administration

Selected respondent quotes
We are lucky to have support for our program. I also have four program coordinators who teach and precept; they each have 0.5 FTE dedicated to the fellowship.
Our program director role is 0.5 FTE clinical and 0.5 FTE for the program, which includes administration and teaching. We have x2 program co-directors.
We have two dedicated program staff for our multi-track program, as well as a staff member hired for an HRSA grant. The program director is non-clinical but works on a wide variety of additional work/projects including oversight of the NP residency programs.
We have a multi-site, multi-specialty track. My administrative duties are not tied directly to a speciﬁc track but rather to overseeing all tracks.
Our (APP) fellowship does not have a program director. Our organization does not have dedicated staff for the (APP) fellowship, but staff assigned to fellowship duties as part of their scope of work.
Being the program coordinator is one of my many duties; there is no dedicated staff for the program. Though the institution wants the increased funding a residency program brings in, they are slow to respond to requests for space for learners, support staff in organizing orientation materials, and provide little to no training for new program directors (learn by trail/error).

Irregular work schedule

According to the survey, most program directors (66%; 96/138) spend four to nine additional hours outside of their scheduled hours on program activities, while 19% (27/138) reported spending 10 or more additional hours on program activities. According to one respondent, “As an NP program director, the FTE configuration is unbalanced, and the director should have a full-time NP coordinator and dedicated paid faculty and support staff for adequate program support."

Program director compensation

**Figure 4:**
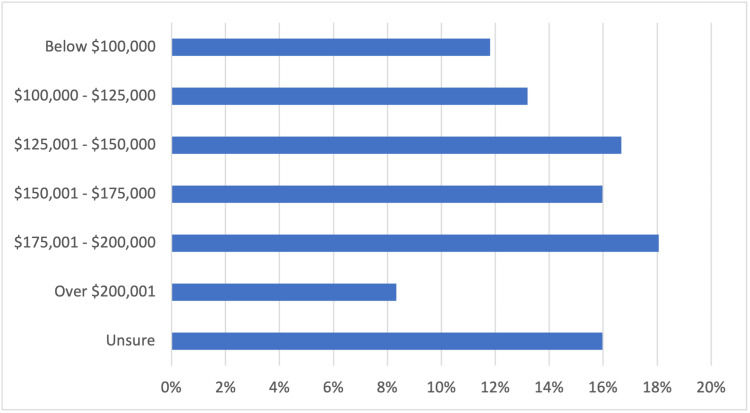
Program director compensation Total respondents: 144

Overall, 42% (60/144) of program directors received $150,000 or less in total compensation (salary, fringe benefits, and bonus incentive payments), whereas 42% (61/144) received more than $150,000 in total compensation. Sixteen percent (23/144) were unsure about their total compensation. We did not investigate whether program director total compensation was influenced by other factors such as geography, years in clinical practice, or employer type. Figure 4 below provides a breakdown of program director total compensation.

Likert analysis results

The total number of respondents who completed Q1-Q4 in this section of the survey was 134 (87.6%). Respondents who did not answer at least one of the four questions of interest (Q1-Q4) were excluded from further analysis. Table 2 presents the frequency counts of missing responses for Q1-Q4.

**Table 2:** Frequency counts of missing responses for Likert questions 1-4

Counts of missing responses	N	%
0	134	87.6
1	1	0.7
2	1	0.7
3	1	0.7
4	1	0.7
6	15	9.8

**Figure 5:**
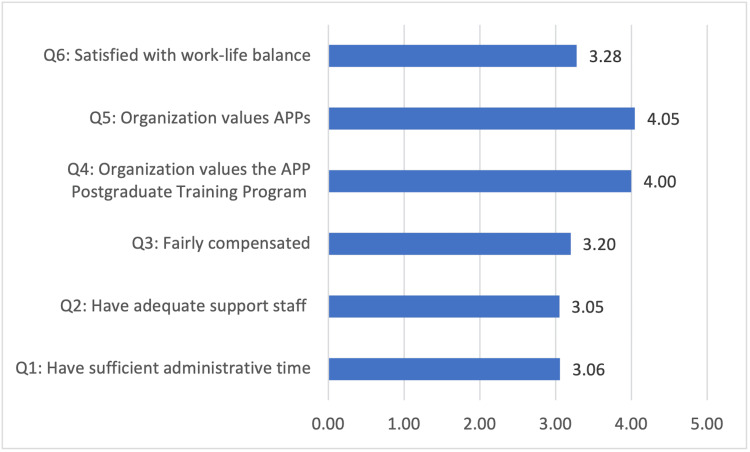
Mean response scores Q1: question 1, Q2: question 2, Q3: question 3, Q4: question 4, Q5: question 5, Q6: question 6

Figure 5 below provides a summary of survey mean response scores. In general, respondents were neither agreeing nor disagreeing regarding having sufficient administrative time (M = 3.06, SD = 1.21), having adequate support staff (M = 3.05, SD = 1.27), and being fairly compensated (M = 3.20, SD = 1.16) for postgraduate program roles and responsibilities. Respondents moderately agreed that their organization values the postgraduate training program (M = 4.00, SD = 1.00) and the APPs (M = 4.05, SD = 0.97). Respondents were neither agreeing nor disagreeing regarding having work-life balance (M = 3.28, SD = 1.11).

The Shapiro-Wilk normality test was used to determine the normality of the data for the dependent and independent variables. A p-value < 0.05 for the Shapiro-Wilk test suggests that the data are not normally distributed (Field, 2013). According to the results of Shapiro-Wilk normality tests (Table 3), the data for all dependent and independent variables were not normally distributed (p < 0.001) for having sufficient administrative time, having adequate support staff, being fairly compensated, and having the organization value the postgraduate program.

**Table 3:** Shapiro-Wilk normality test W: Shapiro-Wilk test statistic, df: degrees of freedom, p: p-value * represents significant p-value < 0.001

Shapiro-Wilk normality test	Results		
	W	df	p
Have sufficient administrative time	0.872	134	(< 0.001*)
Have adequate support staff	0.886	134	(< 0.001*)
Fairly compensated	0.885	134	(< 0.001*)
Organization values the APP postgraduate training program	0.797	134	(< 0.001*)

Since the data were not normally distributed, Spearman's rank correlation coefficients were performed to determine the relationship between the dependent variables and the independent variable (Table 4).

**Table 4:** Spearman’s rank order correlation and p-value APP: advanced practice provider * represents a significant p-value

	Organization "values" the APP postgraduate program
Have sufficient administrative time	0.342 (< 0.001*)
Have adequate support staff	0.340 (< 0.001*)
Fairly compensated	0.356 (< 0.001*)

There was a statistically significant positive relationship between the organization values the postgraduate program and having sufficient administrative time (rs = 0.342, p < 0.001), having adequate support staff (rs = 0.340, p < 0.001), and being fairly compensated (rs = 0.356, p < 0.001) for the postgraduate program role and responsibilities. In other words, if the sponsoring organization valued the postgraduate program, the more likely the respondent indicated having sufficient compensation and program support.

## Discussion

To the best of our knowledge, this is the largest study to depict program directors' perspectives on administrative time and support available in their postgraduate programs. Moreover, this study provides a snapshot of the program director's total compensation as reported by respondents from multiple medical and surgical specialties. In addition, respondents were either from an NP, PA, or joint NP/PA program. In our study, the majority of program directors have five years or more of clinical experience before assuming the role of director, but less than a third have been in their position for at least five years. Moreover, 42% of program directors earn a total compensation of $150,000 or less annually, whereas 42% earn over $150,000 a year. Only 50% of respondents believed they were fairly compensated for their position, and most 66% (96/138) spent four to nine additional hours outside of their scheduled hours on program activities. We did not investigate whether the program director's total compensation was at or above the market rate.

The question of whether extra hours worked outside of scheduled hours were related to oversight of the curriculum, program assessment, applicant recruitment events, scheduled clinical shifts, counseling and mentoring of trainees, and other learners is unknown. Also, whether program directors received additional compensation for hours worked outside of scheduled hours was not part of this study. Nevertheless, the results of this study provide insight into the total compensation received by program directors.

In terms of program support, the distribution of full-time support personnel varied across postgraduate programs. Slightly less than half of respondents reported having adequate support staff and sufficient administrative time to address program responsibilities. Consequently, 52% (68/133) of respondents have a total of 0.5 FTE or less for administrative (non-clinical) duties related to program administration, which is consistent with a prior study [1]. Additionally, less than half of the respondents indicated that they were satisfied with their work-life balance. Though the causes of poor work-life balance are multi-factorial, having insufficient administrative time, support, and irregular work schedules have been partly implicated in perceived dissatisfaction with work-life balance among physician program directors [13,14].

Among accredited programs, slightly more than half of respondents felt that they had sufficient administrative time and adequate support to address programmatic responsibilities. Although the majority of postgraduate programs in this study are unaccredited, the impact of accreditation remains an active area of research.

Despite these novel findings, many questions remain as to what types of resources, staff, and dedicated time are required to facilitate APP postgraduate program administration and operations. Our study underscores the need for national guidelines around the appropriate level of program support to facilitate specialty training based on program size. Unfortunately, there are no publicly available datasets regarding an FTE personnel model for APP residency and fellowship postgraduate programs in the United States. Moreover, there are neither standardized requirements across APP accreditors nor any defined minimum dedicated time for program administration. Additionally, program director roles and responsibilities vary greatly among institutions, and most published research has examined or highlighted the roles and responsibilities of program directors in a single medical specialty [11,15-17]. To date, there are no large studies that clearly characterize the responsibilities and roles of directors and other administrative personnel across various specialties and program types. Therefore, APP accreditors must better codify the importance and requirements of having sufficient staffing and resources that support the wide range of administrative functions within postgraduate programs.

Areas of future research

Further research is needed to investigate administrative staffing levels and funding sources across different single-track versus multi-track postgraduate training program specialties to better assess the impact of these variables on educational quality and program sustainability. Another area of interest is whether years of clinical experience as a program director or oversight of single versus joint postgraduate programs are directly correlated with a higher total compensation structure. In addition, further research is necessary to understand the administrative staff FTEs allotted to accredited postgraduate programs versus non-accredited programs of similar size. Lastly, research is needed to examine which departmental and institutional factors impact program director turnover.

Limitations

Despite these valuable and unparalleled insights, this study is not without limitations. First, this study utilized a cross-sectional design and thus does not allow for causal inferences. Second, although the response rate was relatively low, this is the largest survey of postgraduate programs to date. The low response rate limited the generalizability of the study results and the ability to detect significant differences between respondents and nonrespondents. Also, the survey was pretested but not validated, and some respondents may have had difficulty interpreting questions, which may have led to some incomplete survey responses. Further, PA postgraduate programs were underrepresented in our study. What's more, postgraduate midwifery fellowships, which are few in number, did not participate in our study. Lastly, given the anonymous nature of the electronic survey, it is possible that a respondent may have answered more than once. Despite these limitations, this study has provided a unique insight into the current state of operational support available in many APP postgraduate programs to facilitate specialty training, including information regarding program director compensation.

## Conclusions

This study summarizes the current state of personnel support present in many APP postgraduate programs, along with program director compensation data. Based on the study findings, there is a need for research and guidance to ensure that postgraduate programs have sufficient personnel, protected time, and financial support to fulfill administrative responsibilities. Additionally, more research is needed across various specialties to better understand the administrative tasks associated with the role of program directors and other administrative personnel within APP postgraduate programs.
